# 627 Use of Tubular Elastic Bandage to Reduce Device Related PI (PI) in Burn Patients

**DOI:** 10.1093/jbcr/iraf019.256

**Published:** 2025-04-01

**Authors:** John Hiller, Cristina Moran, Katy Newton

**Affiliations:** Burn Care Center at MetroHealth; MetroHealth; MetroHealth

## Abstract

**Introduction:**

Patients admitted with significant burn injuries often require the use of restrictive dressings, therapeutic elastic wraps and specialized wound care devices, all of which can result in complications including edema and excess drainage. These necessary wound care practices can increase the risk of PI due to hemodynamic instability, mobility issues, reduced or poor perfusion, and the use of additional pharmacologic agents. Device related PI are estimated to account for almost 30% of PI in all patients. Beyond the potential for poorer patient outcomes, PI are also associated with costly organizational level impacts including increased intensive treatment(s) and pain management, longer lengths of stay, and possible infection. Currently, little evidence exists on best practices in PI reduction in the care of burn patients. However, we argue that alternative dressings, including tubular elastic bandages which can be measured to fit the patient in order to provide compression, reduce edema and reduce the risk for PI, ought to be considered as a medically effective and less costly option to use for wound dressing care in the burn patients.

**Methods:**

The setting is an 8 bed Burn Intensive Care Unit in a large, urban, academic public health system designated as a Level I trauma and burn center. Patient population includes adults and pediatric patients with a wide variety of burn injuries. Twenty five nursing staff were educated on interventions to reduce device related injury including properly sizing of tubular elastic bandages with return demonstration of proper technique. Additionally, unit champions created a video of proper dressing placement which was viewed by all burn unit staff. Dressings were made readily available near burn treatment rooms in a variety of sizes. Weekly skin rounds were completed by the unit charge nurses, nurse manager and Clinical EBP Specialist to assess areas at risk for PI under devices and adherence to tubular elastic bandage dressing.

**Results:**

In 2023, 50% of PI in the unit were related to ace wraps and devices. There were inconsistencies in dressing types and staff surveyed were unaware of alternate dressings and the appropriate sizing and measuring techniques. There have been zero incidences of PI in the BICU since implementation in Dec 2023.

**Conclusions:**

There is increased compliance when interventions are clearly defined with continued surveillance of risk for PI. The project supported the use alternative dressings, including tubular elastic bandages which can be measured to fit the patient in order to provide compression, reduce edema and reduce the risk for PI, ought to be considered as a medically effective and less costly option to use for wound dressing care in the burn patients.

**Applicability of Research to Practice:**

Continued surveillance and formal research has potential to examine additional PI prevention measures in the burn population.

**Funding for the Study:**

N/A

